# Peripheral blood‐derived immune cell counts as prognostic indicators and their relationship with DNA methylation subclasses in glioblastoma patients

**DOI:** 10.1111/bpa.13334

**Published:** 2025-02-03

**Authors:** Benedikt Asey, Tobias F. Pantel, Malte Mohme, Yahya Zghaibeh, Lasse Dührsen, Dana Silverbush, Ulrich Schüller, Richard Drexler, Franz L. Ricklefs

**Affiliations:** ^1^ Department of Neurosurgery University Medical Center Hamburg‐Eppendorf Hamburg Germany; ^2^ Department of Cancer Biology, Perelman School of Medicine University of Pennsylvania Philadelphia Pennsylvania USA; ^3^ Abramson Family Cancer Research Institute, Perelman School of Medicine University of Pennsylvania Philadelphia Pennsylvania USA; ^4^ Institute of Neuropathology University Medical Center Hamburg‐Eppendorf Hamburg Germany; ^5^ Department of Pediatric Hematology and Oncology, Research Institute Children's Cancer Center Hamburg University Medical Center Hamburg‐Eppendorf Hamburg Germany; ^6^ Research Institute Children's Cancer Center Hamburg Hamburg Germany

**Keywords:** composite score, glioblastoma, immune, methylation, outcome, prognosis

## Abstract

Glioblastomas are known for their immunosuppressive tumor microenvironment, which may explain the failure of most clinical trials in the past decade. Recent studies have emphasized the significance of stratifying glioblastoma patients to predict better therapeutic responses and survival outcomes. This study aims to investigate the prognostic relevance of peripheral immune cell counts sampled prior to surgery, with a special focus on methylation‐based subclassification. Peripheral blood was sampled in patients with newly diagnosed (*n* = 176) and recurrent (*n* = 41) glioblastoma at the time of surgery and analyzed for neutrophils, monocytes, leukocytes, platelets, neutrophil–lymphocyte ratio, lymphocyte–monocyte ratio, and platelet–lymphocyte ratio. Peripheral immune cell counts were correlated with patients' survival after combined radiochemotherapy. In addition, 850 k genome‐wide DNA methylation was assessed on tissue for defining tumor subclasses and performing cell‐type deconvolution. In newly diagnosed glioblastoma, patients with higher peripheral neutrophil counts had an unfavorable overall survival (OS) (*p* = 0.01, median overall‐survival (mOS) 17.0 vs. 10.0 months). At the time of first recurrence, a significant decrease of peripheral immune cell counts was observed, and elevated monocyte (*p* = 0.03), neutrophil (*p* = 0.04), and platelet (*p* = 0.01) counts were associated with poorer survival outcomes. DNA methylation subclass‐stratified analysis revealed a significant survival influence of neutrophils (*p* = 0.007) and lymphocytes (*p* = 0.04) in the mesenchymal (MES) subclass. Integrating deconvolution of matched tumor tissue showed that platelets and monocytes were correlated with a more differentiated, tumor‐progressive cell state, and peripheral immune cell counts were most accurately reflected in tissue of the MES subclass. This study illustrates a restricted prognostic significance of peripheral immune cell counts in newly diagnosed glioblastoma and a constrained representation in matched tumor tissue, but it demonstrates a more pertinent situation at the time of recurrence and after DNA methylation‐based stratification.

## Graphical abstract

This study illustrates the prognostic relevance of epigenetic molecular profiling and the peripheral immune cell counts in newly diagnosed and matched recurrent glioblastoma patients.

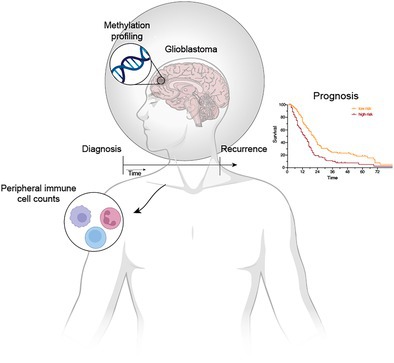

## 1 INTRODUCTION

The optimal management of isocitrate dehydrogenase (IDH)‐wildtype glioblastoma presents a formidable challenge because of its infiltrative and highly aggressive nature [1, 2]. The current standard treatment involves maximal safe resection followed by adjuvant combined radiochemotherapy for newly diagnosed glioblastoma [3, 4]. Despite the multimodal therapeutic approach, long‐term local tumor control remains critical, with only rare cases of success, while most patients experience recurrence [5, 6]. In response to this daunting prognosis and the limited therapeutic responses, immunotherapies have assumed a prominent role in the field of neuro‐oncology [7, 8]. The increasing understanding of pivotal drivers within the immune microenvironment has catalyzed this shift in focus [8, 9]. It was also shown that immune cell infiltrates are systematically detectable in tumor tissue in different concentrations and profiles. In addition to that, the distribution of immune cells in the microenvironment of patients with glioblastoma also appears to be a decisive factor for the outcome of patients. It was shown that patients with mixed myeloid and T‐lymphocytic infiltrates have a significantly worse outcome [10].

On the other hand, it has been shown that the presence of glioma‐associated macrophages or monocytes in tumor tissue appears to have a favorable influence on the survival of patients with glioblastoma [11]. Because of these facts, numerous ongoing trials are endeavoring to unlock the potential benefits of understanding the connection between tumor growth and immune response [12].

However, it is noteworthy that most clinical trials have failed to meet expectations [13]. Nevertheless, post hoc analyses of recent immunotherapy trials have successfully identified subgroups of glioblastoma patients exhibiting exceptional therapeutic responses, resulting in significant extensions of survival [14, 15, 16].

These findings underscore the critical importance of stratifying glioblastoma patients based on their tumor subtypes through RNA‐sequencing [17] or DNA methylation [18, 19]‐based methods. Ideally, subtype determination could be performed using patients' blood or cerebrospinal fluid.

One topic of discussion concerning such stratification is the utilization of immune cell counts in peripheral blood, a routine procedure in most glioblastoma patients. Previous studies have shown the potential of using peripheral blood to determine immune profiles [20] and predict patients' survival in newly diagnosed glioblastoma [21, 22, 23]. However, the identification of the most suitable immune cell for predicting survival has yielded differing results among these studies, and a few have even presented contradictory findings [21, 24].

One possible explanation for this inconsistency in data across various cohort studies could be the absence of stratification based on glioblastoma subtypes within the tumor tissue. An emerging field involves the DNA methylation‐based classification of central nervous system tumors [18, 25], allowing for the subclassification of glioblastomas into six distinct categories [19], with receptor tyrosine kinase (RTK) I, RTK II, and mesenchymal (MES) being the most prevalent. Recent research has demonstrated variable responses to surgery [26] and immunotherapy [27] across these subclasses, prompting questions about how these subclasses manifest in peripheral immune cell counts and whether further subclassification can enhance the predictive capacity of these immune cells.

The primary objective of this study was to assess the prognostic significance of preoperative peripheral immune cell counts in both newly diagnosed and recurrent glioblastoma, with a particular emphasis on DNA methylation‐based subclassification and blood‐tissue matched deconvolution analysis.

## 2 METHODS

### 2.1 Study population

Patients undergoing surgery with the diagnosis of isocitrate‐dehydrogenase (IDH)‐wildtype glioblastoma were identified from a prospectively collected and maintained database of the Department of Neurosurgery at the University Medical Center Hamburg‐Eppendorf, Germany. Patients were included when receiving adjuvant combined radiochemotherapy after Stupp [3] or CeTeG [28] regimen and with a follow‐up time of at least 3 months after surgery. Data collection, including patient follow‐up, was completed on December 31, 2023. Informed written consent was obtained from all patients. Diagnosis was based on the current World Health Organisation (WHO) classification for central nervous tumors (WHO 2021) [29]. The extent of resection was stratified into gross total resection (GTR), near GTR, partial resection (PR), and stereotactic biopsy. A GTR was defined as a complete removal of contrast‐enhancing parts, a near GTR as a removal of more than 90% of the contrast‐enhancing parts, whereas a resection of lower than 90% was defined as PR. The extent of resection of contrast‐enhancing parts was evaluated in the magnetic resonance imaging (MRI) up to 48 h after surgery. Overall survival (OS) was measured from the date of surgery to the date of death from any cause, while progression‐free survival (PFS) was measured from the date of surgery to the occurrence of the first progression. Additionally, progression‐to‐overall survival (POS) was calculated from the date of recurrence surgery to the date of death from any cause. A progress was defined according to Response Assessment in Neuro‐Oncology (RANO) criteria [30]. All patients included from the initial surgery were screened for potential re‐resection at the time of first local recurrence. Eligibility for recurrence surgery was determined by clinical decision‐making of the local tumor board. Patients who underwent re‐resection had blood samples taken prior to the re‐resection, which were then analyzed and matched to their blood profiles from the initial surgery. Dexamethasone usage was recorded for each patient to assess their daily dosage intake. Patients with chronic dexamethasone treatment prior to their glioblastoma diagnosis were excluded from this study.

### 2.2 Peripheral blood‐derived immune counts and composite scores

Consecutive patients were included if preoperative neutrophil, lymphocyte, platelet, and monocyte counts were available. Preoperative neutrophil, lymphocyte, platelet, and monocyte counts were obtained from a blood withdrawal within 3 days prior to the first surgery. All samples were collected before the administration of surgery‐related medication. The composite scores neutrophil–lymphocyte ratio (NLR), platelet–lymphocyte ratio (PLR), and lymphocyte–monocyte ratio (LMR) were all calculated by directly dividing the former by the latter.

### 2.3 DNA methylation profiling

DNA was extracted from tumor tissue and analyzed for genome‐wide DNA methylation patterns using the Illumina Infinium MethylationEPIC array (EPIC)  v2 (850 k). For bioinformatic reasons, converted data to the 450 K array format was used when conducting deconvolution analysis to determine cell type compositions, as previously described [31, 32]. Processing of DNA methylation data was performed with custom approaches [25]. Methylation profiling results from the first surgery were submitted to the molecular neuropathology (MNP) methylation classifier v12.8 hosted by the German Cancer Research Center (DKFZ) [18]. Patients were included if the calibrated score for the specific methylation class was >0.84 at the time of diagnosis in accordance with recommendations by Capper et al. [25] For *IDH*‐wildtype glioblastoma, patients with a score below 0.84 but above 0.7 with a combined gain of chromosome 7 and loss of chromosome 10 or amplification of epidermal growth factor receptor (*EGFR*) were included in accordance with consortium to inform molecular and practical approaches to CNS tumor taxonomy (cIMPACT‐NOW) criteria [33]. Furthermore, a class member score of ≥0.5 for one of the glioblastoma subclasses was required. Evaluation of the *MGMT* promoter methylation status was made from the classifier output v12.8 using the *MGMT*‐STP27 method [34].

### 2.4 Processing of methylation arrays

All idats corresponding to methylation array data were processed similarly using the minfi package in R (version 1.40.0) [35]. The data was processed using the preprocessIllumina function. Only probes with detection *p*‐values <0.01 were kept for further analysis. Also, probes with <3 beads in at least 5% of samples, as well as all non‐5'‐Cytosine‐phosphate‐Guanine‐3'(CpG) probes, single nucleotide polymorphism (SNP)‐related probes, and probes located on X and Y chromosomes were discarded. The CpG intensities were converted into beta values representing total methylation levels (between 0 and 1).

### 2.5 Cell state composition analysis

To infer the abundance of cell type and cell state in the samples, we subjected each sample to a bulk DNA methylation assay using EPIC arrays and applied the Silverbush et al. deconvolution method [36]. The deconvolution method is a reference‐free method that uses a hierarchical matrix factorization approach inferring both cell types and the cell states therein. The method was trained on the DKFZ glioblastoma cohort and tested on the The Cancer Genome Atlas (TCGA) glioblastoma cohort and was able to infer the abundance of cell types in the microenvironment (immune, glia, and neuron) and malignant cell states (malignant stem‐like cells component and two differentiated cells components). We applied the method as described in Silverbush et al. using the cell type and cell state encoding provided in the manuscript and via the engine provided in EpiDISH [37] package, with the robust partial correlation (RPC) method and a maximum iterations of 2000.

### 2.6 Cell type deconvolution

Non‐negative least square (NNLS) linear regression was used in deconvolving the beta values of methylation arrays into cell type components [38, 39, 40]. As a reference, a publicly available signature was obtained from Moss et al. consisting of gene expressions for 25 cell type components (Monocytes_EPIC, B‐cells_EPIC, CD4T‐cells_EPIC, NK‐cells_EPIC, CD8T‐cells_EPIC, Neutrophils_EPIC, Erythrocyte_progenitors, Adipocytes, Cortical_neurons, Hepatocytes, Lung_cells, Pancreatic_beta_cells, Pancreatic_acinar_cells, Pancreatic_duct_cells, Vascular_endothelial_cells, Colon_epithelial_cells, Left_atrium, Bladder, Breast, Head_and_neck_larynx, Kidney, Prostate, Thyroid, Upper_GI, and Uterus_cervix) and 6105 unique CpGs [38].

### 2.7 3D volumetric segmentation

We analyzed T1‐weighted as well as T2‐weighted fluid attenuated inversion recovery (FLAIR) MRI axial images of all glioblastoma patients before surgery. The program BRAINLAB was used for all analyses. To measure tumor volume, the tumor region of interest was delineated with the tool “Smart Brush” in every slice by hand, enabling a multiplanar 3D reconstruction. With this methodology, the volume of contrast enhancement and of FLAIR hyperintensity was assessed in cubic centimeter.

### 2.8 Statistical analysis

The data collection, including the patient follow‐up, was completed on December 31, 2023. After that, the differences in continuous variables were analyzed with the Mann–Whitney *U* test, and differences in proportions were analyzed with the chi‐square test or Fisher exact test. Overall and disease‐free survival was evaluated with the Kaplan–Meier method. Univariate and multivariate Cox proportional hazards models were used to assess the effects of variables on seizure outcome and to compute odds ratio (OR). A two‐sided *p*‐value less than 0.05 was considered as statistically significant. All analyses were performed using SPSS Inc. (Chicago, IL, USA).

## 3 RESULTS

In this study, we conducted an analysis of a cohort comprising 176 patients who underwent surgery for newly diagnosed glioblastoma, with preoperative assessments of peripheral immune cell counts. Therefore, we conducted genome‐wide DNA methylation analysis on all 176 samples obtained from the initial diagnosis and subclassified patients based on their DNA methylation subclass, namely RTK I, RTK II, or MES.

Following the initial diagnosis, all patients received radiochemotherapy as an adjuvant treatment. The basic characteristics of the cohort are presented in Table SS1. In brief, the mean age at the time of diagnosis was 63.6 years, and 71 patients (40.3%) were of the female sex. Most of the tumors were localized within the parietal lobe (*n* = 97, 55.1%), followed by the temporal lobe (*n* = 69, 39.2%). Of all tumors, 120 tumors (69.8%) were located within eloquent regions (Table SS1). A GTR was successfully achieved in 65 patients (36.9%), while 82 tumors (46.6%) had a methylated *MGMT* promoter (Table SS1).

### 3.1 A higher peripheral neutrophil count is associated with unfavorable overall survival in newly diagnosed glioblastoma

To address the question of whether peripheral immune cell counts (neutrophils, lymphocytes, platelets, and monocytes) or composite scores (NLR, PLR, and LMR) can serve as predictive markers for the prognosis of glioblastoma patients, we conducted a comprehensive survival analysis involving all 176 patients mentioned in this study. All patients underwent combined radiochemotherapy following surgery and had a minimum follow‐up period of 3 months. As of the final data collection, 137 (77.8%) patients were dead (Figure S1).

Given the existing discord in the literature concerning the impact of dexamethasone on immune cell functionality and counts [41, 42, 43, 44], we initially divided the study population into two cohorts: one comprising patients who received dexamethasone before the first surgery and blood collection and the other consisting of those who did not (Figure S2). In this initial analysis, no significant differences were observed with respect to immune cell counts and composite scores between these two subgroups (Figure S2). Consequently, we decided to include all patients in our survival analysis, regardless of their preoperative dexamethasone treatment, as no survival difference was observed in the multivariate analysis (Table SS2). As the further correlation between patient survival and continuous immune cell counts did not yield significant results (Table SS3), patients were subsequently categorized based on the median value of each immune cell count, as detailed in Table SS4. Further correlation with additional clinical features revealed lower platelet levels in older patients (Figure S3) but no other associations with additional clinical variables (Figures S4 and S5).

**FIGURE 1:**
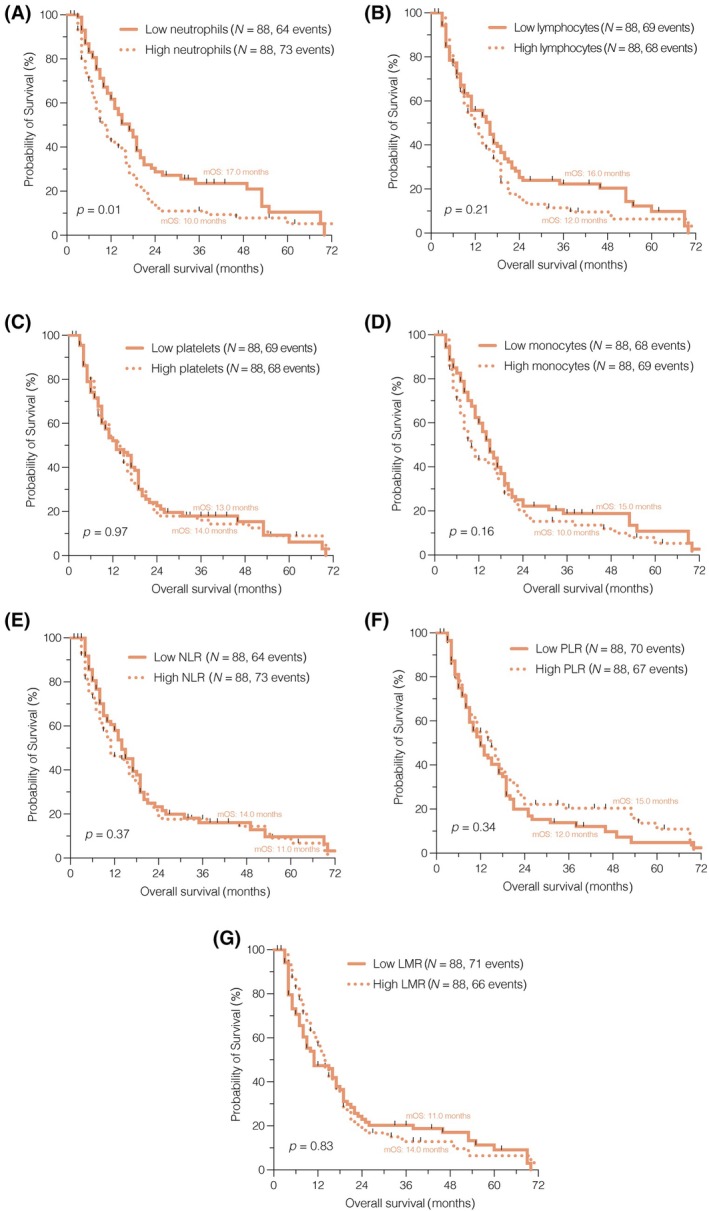
Survival analysis on patients with newly diagnosed glioblastoma, comparing low and high immune cell counts as well as the composite scores. LMR, lymphocyte–monocyte ratio; NLR, neutrophil–lymphocyte ratio; PLR, platelet–lymphocyte ratio.

The results of our survival analysis unveiled a notably improved OS among patients with lower peripheral neutrophil counts (*p* = 0.01, median OS 17.0 vs. 10.0 months; Figure 1A). However, none of the other immune cell counts (Figure 1B–D) or composite scores (Figure 1E–G) demonstrated prognostic significance in the context of newly diagnosed glioblastoma when dichotomized by the median (Table SS4).

### 3.2 Peripheral immune cells are decreased at the time of progression and predict overall survival in recurrent glioblastoma

**FIGURE 2:**
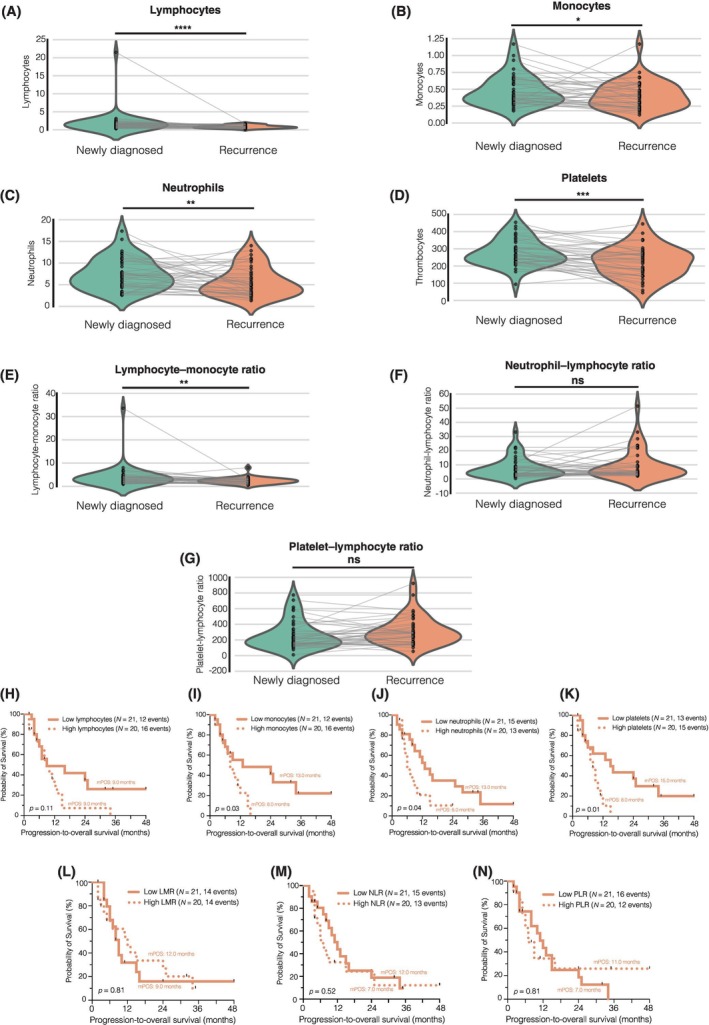
(A–G) Comparison of peripheral immune cell counts and composite scores between newly diagnosed and recurrent glioblastoma in 41 matched patients. (H–N) Influence of peripheral immune cell counts and composite scores on progression‐to‐overall survival after recurrent surgery in 41 patients. LMR, lymphocyte–monocyte ratio; NLR, neutrophil–lymphocyte ratio; PLR, platelet–lymphocyte ratio. ns *p* > 0.05, *p < 0.05, **p < 0.01, ***p < 0.001, ****p < 0.0001.

In our study, a total of 176 patients were included, of which 41 underwent re‐resection for local recurrent glioblastoma following the completion of combined radiochemotherapy. For these patients, peripheral immune cell counts were obtained 3 days prior to the re‐resection. We conducted a detailed analysis of immune cell counts and composite scores in this matched‐pair patient cohort (Figure 2A–G). At the time of re‐resection, significant alterations were observed in various blood parameters. Specifically, lymphocytes (*p* = 0.00004, Figure 2A), monocytes (*p* = 0.0435, Figure 2B), neutrophils (*p* = 0.0078, Figure 2C), and platelets (*p* = 0.00016, Figure 2D) exhibited a marked decrease. Additionally, the LMR showed a significant reduction (*p* = 0.0096, Figure 2E). However, no significant differences were observed in the NLR (*p* = 0.925, Figure 2F) and the PLR (*p* = 0.155, Figure 2G) when compared with their counterparts with newly diagnosed glioblastoma when dichotomized by the median.

All patients were followed up after re‐resection with 28 patients (68.3%) were reported as dead. The patient cohort was stratified based on the median peripheral immune cell counts at the time of recurrent surgery (Figure 2H–N). Survival analysis revealed an unfavorable prognosis for patients with elevated monocyte (*p* = 0.03; Figure 2I), neutrophil (*p* = 0.04; Figure 2J), and platelet counts (*p* = 0.01; Figure 2K). However, the composite scores (LMR, NLR, and PLR) were found to be insufficient for predicting patients' survival following re‐resection when dichotomized by the median (Figure 2L–N).

Our findings underscore the changes in peripheral immune cell counts at the time of first recurrence after combined radiochemotherapy and highlight the association between higher cell counts and poorer survival outcomes in these patients.

### 3.3 Integrated DNA methylation analysis alters the predictive potential of peripheral immune cell counts based on subclass

**FIGURE 3:**
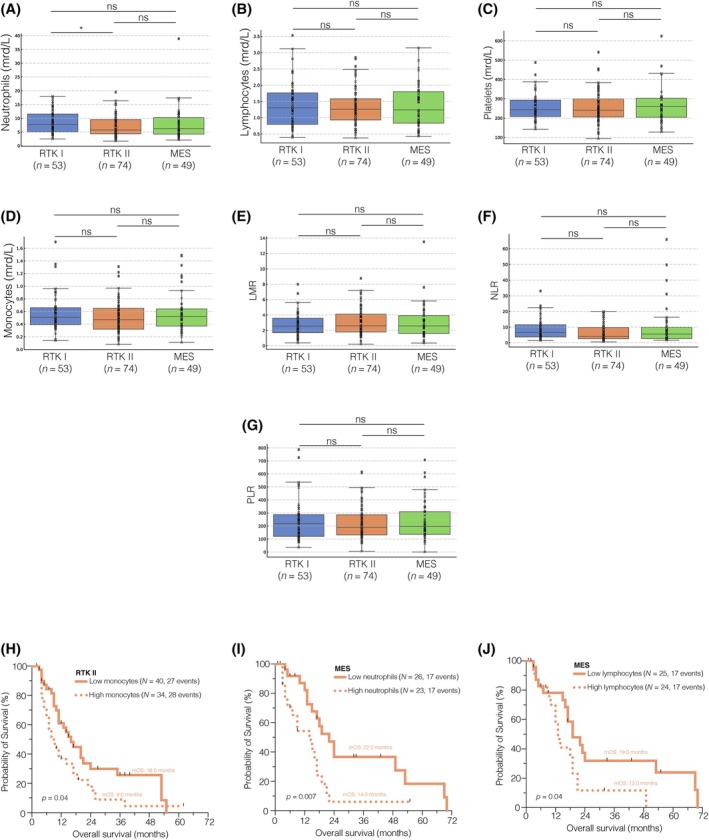
Peripheral immune cell counts, and composite scores stratified to DNA methylation subclasses. (A–G) Comparison of cell counts and composite scores between DNA methylation subclasses. (H–J) Kaplan Meier curves showing statistically significant association within each DNA methylation subclass. ns *p* > 0.05, *p < 0.05. LMR, lymphocyte–monocyte ratio; MES, mesenchymal; NLR, neutrophil–lymphocyte ratio; PLR, platelet–lymphocyte ratio; RTK, receptor tyrosine kinase.

It is evident that there are DNA methylation subclass‐specific signaling pathway aberrations [45], and the response to immunotherapy might be selective for certain subclasses [27]. Therefore, we conducted genome‐wide DNA methylation analysis on all 176 samples obtained from the initial diagnosis and subclassified patients based on their DNA methylation subclass, namely RTK I, RTK II, or MES [18, 19]. The DNA methylation subclass‐stratified comparison of peripheral immune cell counts and composite scores showed significantly higher neutrophil counts in the blood of RTK I patients compared to RTK II patients (*p* = 0.02; Figure 3A). However, all other immune cell counts and composite scores did not differ between the subclasses (Figure 3B–G).

When we performed survival analysis for each DNA methylation subclass using the aforementioned cutoff values (Table SS4), we found a significant influence of monocytes in the RTK II subclass (*p* = 0.04, median OS 16.0 vs. 9.0 months; Figure 3H), and of neutrophils (*p* = 0.007, median OS 22.0 vs. 14.0 months; Figure 3I), as well as lymphocytes (*p* = 0.04, median OS 19.0 vs. 13.0 months; Figure 3J) in the MES subclass on the OS of newly diagnosed glioblastoma patients. All other immune cell counts were not predictive of the OS when dichotomized by the median, regardless of whether classified as RTK I (Figure SS6), RTK II (Figure SS7), or MES (Figure SS8).

In summary, these findings demonstrate comparable peripheral immune cell counts across DNA methylation subclasses but underscore the importance of subclassification when analyzing survival outcomes.

### 3.4 Correlation of peripheral immune cell counts with cell states and infiltrating immune cells in matched tumor tissue

**FIGURE 4:**
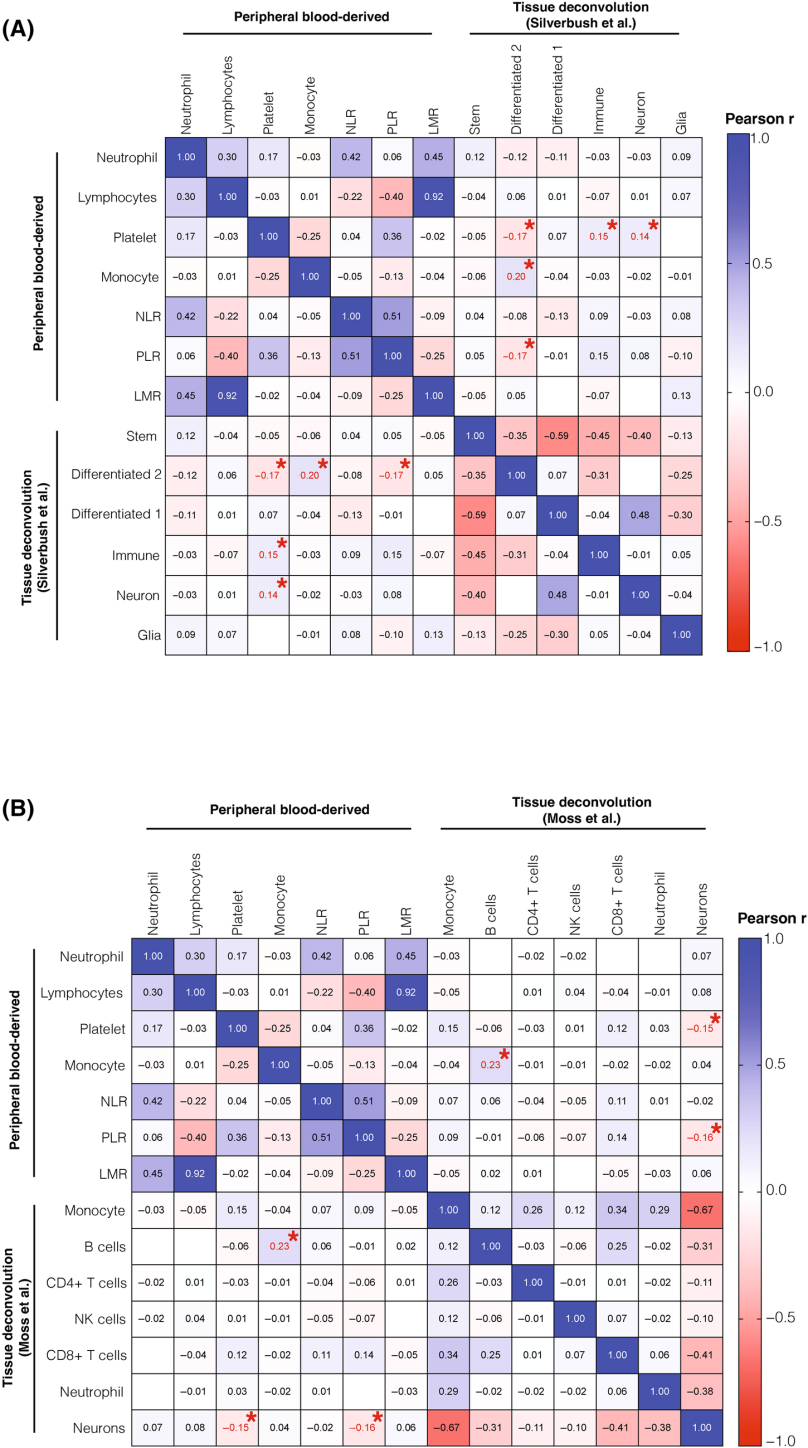
Correlation matrix showing association between peripheral immune blood counts and composites scores with (A) cell states of matched tissue using deconvolution (Silverbush et al.), and (B) immune cells of matched tissue using deconvolution (Moss et al.). *p < 0.05. LMR, lymphocyte–monocyte ratio; NLR, neutrophil–lymphocyte ratio; PLR, platelet–lymphocyte ratio.

Utilizing a deconvolution approach of DNA methylation data has emerged as a powerful tool for defining cell states and the composition of tumor tissue, as demonstrated in previous studies [38, 46]. These analyses have revealed significant enrichment of different cell types between subclasses [47, 48]. We posed the question of whether peripheral immune cell counts are accurately reflected in the cell states and infiltrating cells within the tumor tissue. To investigate this, we performed deconvolution analysis on matched tumor tissue samples from all 176 patients (Figure 4). Using a reference dataset provided by Silverbush et al., we observed a significant association between platelets (*p* = 0.02, *r* = −0.17; Figure 4A), monocytes (*p* = 0.01, *r* = 0.20; Figure 4A), and the PLR (*p* = 0.03, *r* = −0.17; Figure 4A) with a more differentiated, tumor‐progressive cell state (“differentiated‐2”). Additionally, platelets exhibited significant correlations with immune (*p* = 0.04, *r* = 0.15; Figure 4A) and neuron (*p* = 0.04, *r* = 0.14; Figure 4A) signatures. Further, when we delved into a more detailed breakdown of the immune compartment using a reference signature by Moss et al. [38], we found an association between peripheral monocytes and tissue‐infiltrating B cells (*p* = 0.004, *r* = 0.23; Figure 4B). However, it is important to note that all other peripheral immune cell counts did not show a consistent reflection in the tumor tissue deconvolution approach across all tumors (Figure 4B). Upon stratification into DNA methylation subclasses, we observed no significant association between peripheral immune cell counts and tumor tissue‐infiltrating cells in the RTK I (Figure S9A) and RTK II (Figure S9B) subclasses. However, in the MES subclass, especially tumor‐infiltrating B cells, we observed significant correlations (Figure S9C).

## 4 DISCUSSION

Immunotherapies have gained prominence in neuro‐oncology, with ongoing trials seeking to realize their potential, despite past clinical trials falling short. Subgroup analysis of recent immunotherapy trials has revealed exceptional responses in certain glioblastoma patients, emphasizing the need to stratify patients by tumor subclass, including through peripheral blood‐based methods, to improve predictive accuracy. In this study, we determined preoperative peripheral immune cell counts in newly diagnosed and recurrent glioblastoma patients to determine the prognostic relevance and integrated DNA methylation‐based subclassification and blood‐tissue matched deconvolution analysis. In brief, this study contributes the following findings:

(1) Patients with higher peripheral neutrophil counts had an unfavorable OS in newly diagnosed glioblastoma. (2) At the time of progression, there were significant decreases in lymphocytes, monocytes, neutrophils, and platelets in patients with recurrent glioblastoma. Elevated monocyte, neutrophil, and platelet counts at recurrence were associated with poorer survival outcomes. (3) DNA methylation subclass‐stratified analysis revealed significantly higher neutrophil counts in the blood of RTK I patients compared to RTK II patients. Neutrophils and lymphocytes had a significant influence on OS in newly diagnosed MES tumors. (4) Deconvolution analysis of matched tumor tissue samples showed that platelets, monocytes, and the PLR were correlated with a more differentiated, tumor‐progressive cell state, and tissue‐infiltrating B cells were correlated with peripheral monocytes, lymphocytes, the neutrophil–lymphocyte, and PLR in the MES subclass.

Despite extensive efforts in the past decades to enhance the survival of glioblastoma patients and the introduction of potential new therapeutic agents targeting critical immunogenic drivers in the tumor microenvironment, most studies have failed to demonstrate prolonged survival [13]. Consequently, the standard treatment for newly diagnosed glioblastoma remains combined radiochemotherapy following the Stupp regimen [3], with no established standard of care for recurrent glioblastoma [6]. However, integrative multimodal studies have deciphered tumor composition and defined glioblastoma subtypes through RNA [17] or DNA methylation profiling [19], offering hope for stratifying patients based on their tumor profiles and predicting responses to current therapies or future clinical trials. While these subclassifications are typically based on tumor tissue, relying on accurate sampling with sufficient tumor cell content, predicting responders using patients' peripheral blood would be a more elegant approach. One routine approach is determining immune cell counts, including neutrophils, leukocytes, monocytes, and platelets from peripheral blood, from which various ratios can be derived [49]. Recent meta‐analyses have indicated worse OS in patients with a higher NLR [21], and Wang et al. demonstrated a correlation between circulating neutrophils and patient outcomes [23]. However, a closer examination of individual studies reveals conflicting results regarding peripheral immune cell counts [50, 51, 52, 53, 54]. Our study was unable to replicate the prognostic relevance of the NLR, neither at the time of diagnosis nor at the time of recurrence. Nevertheless, peripheral neutrophil cell count emerged as the most predictive factor at both timepoints. The survival difference between patients with low and high peripheral neutrophil counts was even more pronounced in the DNA methylation‐based MES subclass, supporting the exceptional immune‐responsive role of MES glioblastomas [27, 55]. Interestingly, the peripheral neutrophil count did not significantly differ in the MES subclass when compared with the RTK I or RTK II subclasses, but a strong correlation between peripheral neutrophils and tissue B cells was observed. These findings regarding circulating neutrophils align with a recent study that identified unique upregulation of neutrophils in patients' blood compared to healthy controls [20]. Additionally, the exceptional role of neutrophils in both blood and tissue in glioblastoma malignancy and progression has been increasingly recognized in recent years [56, 57, 58, 59]. One study introduced a blood‐based neutrophil dexamethasone index that reflects epigenetic changes in neutrophils in response to dexamethasone treatment [60]. The neutrophil index not only changed in a dose‐dependent manner but also served as a stratification tool for glioblastoma patient survival. Although this underscores the potentially crucial role of neutrophils, it is important to note that our study did not observe a significant difference in peripheral neutrophil counts following dexamethasone treatment. This discrepancy may be because of the lower cumulative and daily median dexamethasone usage in our study or could be influenced by the absence of a methylome approach in measuring peripheral neutrophil counts.

In the context of neutrophils' importance, it was observed that monocyte and platelet counts exhibited significant associations with survival after first recurrence. Furthermore, these counts correlated with a more progressive tumor cell state when analyzing matched tumor tissue obtained at the time of diagnosis through methylation‐based deconvolution. It is important to note, however, that the overall correlation between peripheral circulating immune cells and tumor tissue proved to be suboptimal. This correlation did show improvement following methylation‐based subclassification, with particular emphasis on the MES subclass, which demonstrated a stronger interface between tissue and blood. When considered in conjunction with prior research [55, 61, 62, 63, 64], these findings underscore the potential immunocompetence of MES glioblastomas. Thus, distinguishing MES from RTK subclasses may warrant consideration for predicting therapeutic responses in ongoing and future immunotherapeutic trials.

Our findings differ in several aspects from previously published studies. A central distinction lies in the timing of blood draws and the associated biological variations. Previous studies often focused exclusively on either the preoperative or postoperative phase, potentially leading to skewed results. In our study, blood samples were collected both at initial diagnosis and at recurrence, enabling a more nuanced analysis of dynamic changes over time.

Another factor concerns sample processing. Whereas earlier studies often employed diverse protocols for immune cell isolation and storage, we implemented standardized procedures to ensure the highest possible data consistency. Such methodological differences may account for the partly contradictory findings regarding the prognostic significance of the NLR or specific immune cell populations.

Lastly, the platforms used for DNA methylation analysis played a crucial role. While older studies often relied on low‐resolution arrays, we utilized modern high‐resolution methylation chips, allowing for the detection of finer epigenetic differences among subclasses. This technological advancement not only facilitates more precise subclassification but also enables the identification of subtle immunological patterns that might have been overlooked in earlier analyses.

Our findings emphasize the importance of an integrative analysis combining peripheral immune cell counts, DNA methylation, and tumor subclasses. This methodology enables not only more precise patient selection for immunotherapeutic approaches but also a deeper understanding of tumor‐host interactions in glioblastomas. At the same time, the discrepancies with previous studies underscore the necessity of methodological standardization and longitudinal study designs to fully unravel the prognostic and therapeutic relevance of the peripheral immune cell‐DNA methylation axis.

In summary, this study sheds light on the limited prognostic significance of peripheral immune cell counts in newly diagnosed glioblastoma, but it demonstrates a more pertinent situation at the time of recurrence and after DNA methylation‐based stratification.

## 5 LIMITATIONS

This study has several limitations. First, as a retrospective data analysis, it is inherently less robust than prospective clinical trials, and this should be considered when interpreting the results. Additionally, the study lacks a stratification into training and test sets and does not include an external cohort, which limits the generalizability of the findings. Because of the retrospective nature of this study, not all patient characteristics were available, and potential confounders may exist that were not accounted for in the presented analysis. Furthermore, it is important to note that the cohort undergoing recurrence surgery represents a highly selected group because of their eligibility for this procedure. These findings may also be influenced by dexamethasone usage between the first and recurrence surgeries, which we were unable to control for in this study. Moreover, it must be noticed that for bioinformatic reasons, we converted our data to the 450 K array format when conducting deconvolution analysis to determine cell type compositions. Of course, data is lost as a result, but the robustness of the algorithms has already been demonstrated repeatedly [31, 32].

## AUTHOR CONTRIBUTIONS

BA, TFP, US, LD, RD, FLR for acquisition of data, analysis, and interpretation of data, YZ for bioinformatical analysis, BA, RD, and FLR for statistical analysis and drafting of the manuscript. MM and US for technical and material support. FLR and US for study concept and design, obtainment of funding, and study supervision. DS for acquistion of data, analysis and interpretation of data, DS ran the deconvolution algorithm. All authors read and approved the final manuscript.

## FUNDING INFORMATION

Franz L. Ricklefs received funding from Illumina Inc., Ulrich Schüller was supported by the Fördergemeinschaft Kinderkrebszentrum Hamburg.

## CONFLICT OF INTEREST STATEMENT

The authors declare no conflict of interest.

## Supporting information


**Figure S1.** Patient follow‐up time was analyzed using the reverse Kaplan–Meier method, with time calculated from the first diagnosis. The median follow‐up time was 55 months.

**Figure S2.** Peripheral immune cell counts and composite scores in patients with or without preoperative dexamethasone treatment. ns *p* > 0.05.

**Figure S3.** Peripheral immune cell counts and composite scores correlated with the age of patients at the time of initial diagnosis. LMR, lymphocyte–monocyte ratio; NLR, neutrophil–lymphocyte ratio; PLR, platelet–lymphocyte ratio.

**Figure S4.** Peripheral immune cell counts and composite scores correlated with the tumor location. LMR, lymphocyte–monocyte ratio; NLR, neutrophil–lymphocyte ratio; PLR, platelet–lymphocyte ratio.

**Figure S5.** Peripheral immune cell counts and composite scores correlated with the extent of surgery. LMR, lymphocyte–monocyte ratio; NLR, neutrophil–lymphocyte ratio; PLR, platelet–lymphocyte ratio.

**Figure S6.** Survival analysis on patients with newly diagnosed glioblastoma of the receptor tyrosine kinase I (RTK I) subgroup.

**Figure S7.** Survival analysis on patients with newly diagnosed glioblastoma of the receptor tyrosine kinase II (RTK II) subgroup.

**Figure S8.** Survival analysis on patients with newly diagnosed glioblastoma of the mesenchymal I (MES) subgroup.

**Figure S9.** Correlation matrix showing association between peripheral immune blood counts and composites scores with immune cells of matched tissue using deconvolution (Moss et al.) in the DNA methylation subgroup (A) RTK I, (B) RTK II, and (C) MES. *p < 0.05.

**Table S1.** Basic characteristics of the study population. LMR: lymphocyte–monocyte ratio; MGMT, O6‐methylguanine‐DNA‐methyltransferase; NLR, neutrophil–lymphocyte ratio; PLR, platelet–lymphocyte ratio; SD, standard deviation.

**Table S2.** Multivariate analysis was conducted to evaluate the impact of clinical variables on patients' overall survival.

**Table S3.** Cox univariate model to assess the correlation between continuous immune cell counts and patients' overall survival.

**Table S4.** Cutoff values for each peripheral immune cell or composite score to stratify the study population into low or high counts.

## Data Availability

The data presented in this study can be made available upon reasonable request.
