# Supplementary figures and images for: Peripheral blood‐derived immune cell counts as prognostic indicators and their relationship with DNA methylation subclasses in glioblastoma patients
Source: Brain Pathol. 2025 Feb 3;35(4):e13334. doi: 10.1111/bpa.13334 (PMC12145900; doi:10.1111/bpa.13334)

Supplementary Figure 1

A

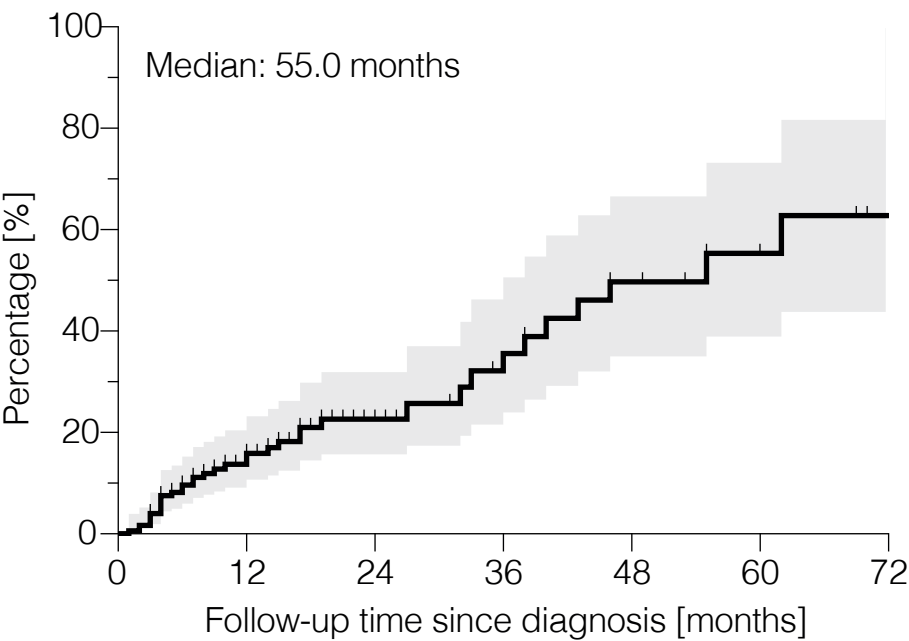

Supplement: Supplementary file 1 — Figure S1. Patient follow‐up time was analyzed using the reverse Kaplan–Meier method, with time calculated from the first diagnosis. The median follow‐up time was 55 months. [file BPA-35-e13334-s008.pdf]

Supplementary Figure 2

**A**

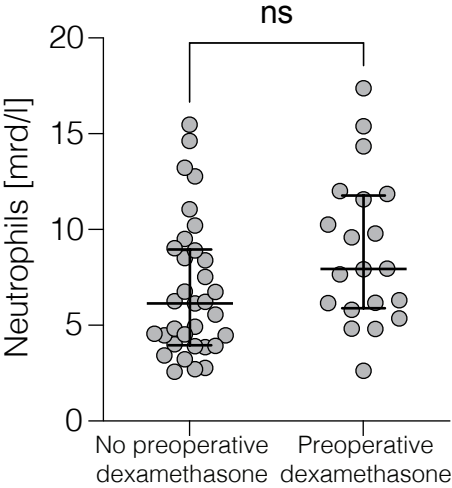

**B**

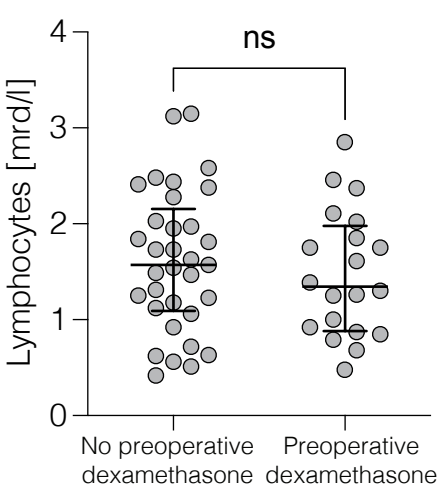

**C**

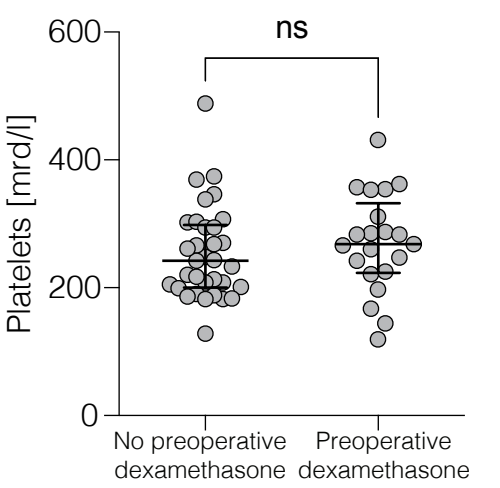

**D**

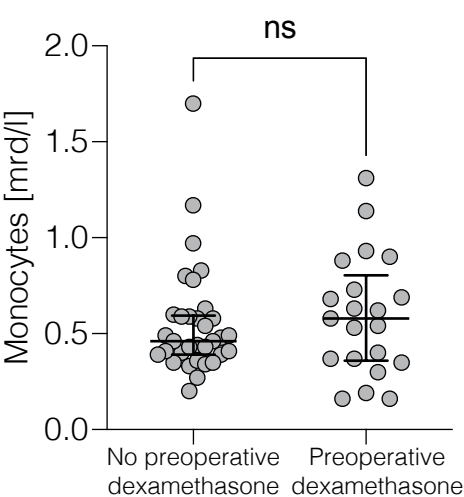

**E**

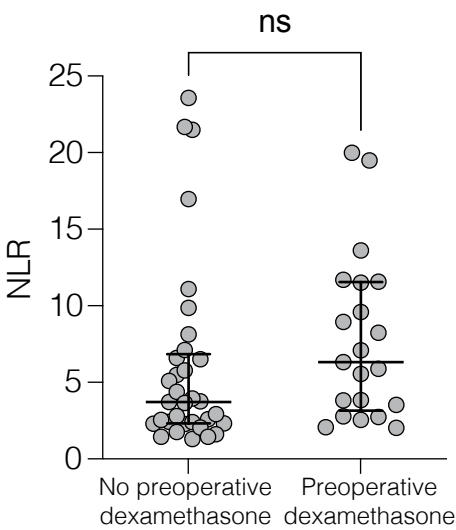

**F**

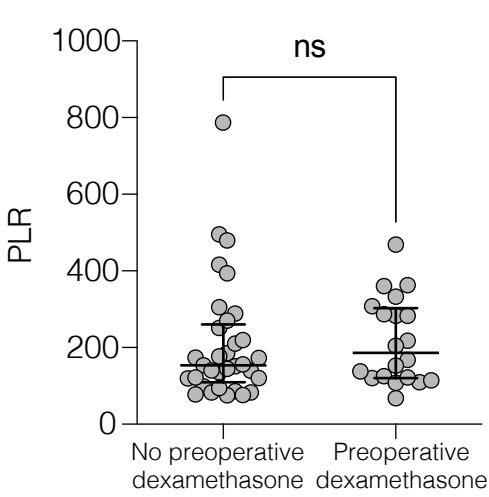

**G**

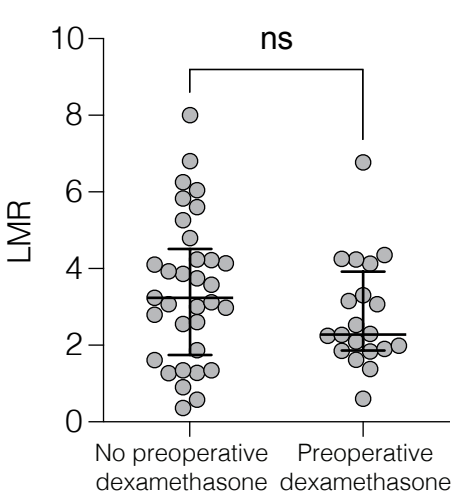

Supplement: Supplementary file 2 — Figure S2. Peripheral immune cell counts and composite scores in patients with or without preoperative dexamethasone treatment. ns p > 0.05. [file BPA-35-e13334-s012.pdf]

# Supplementary Figure 3

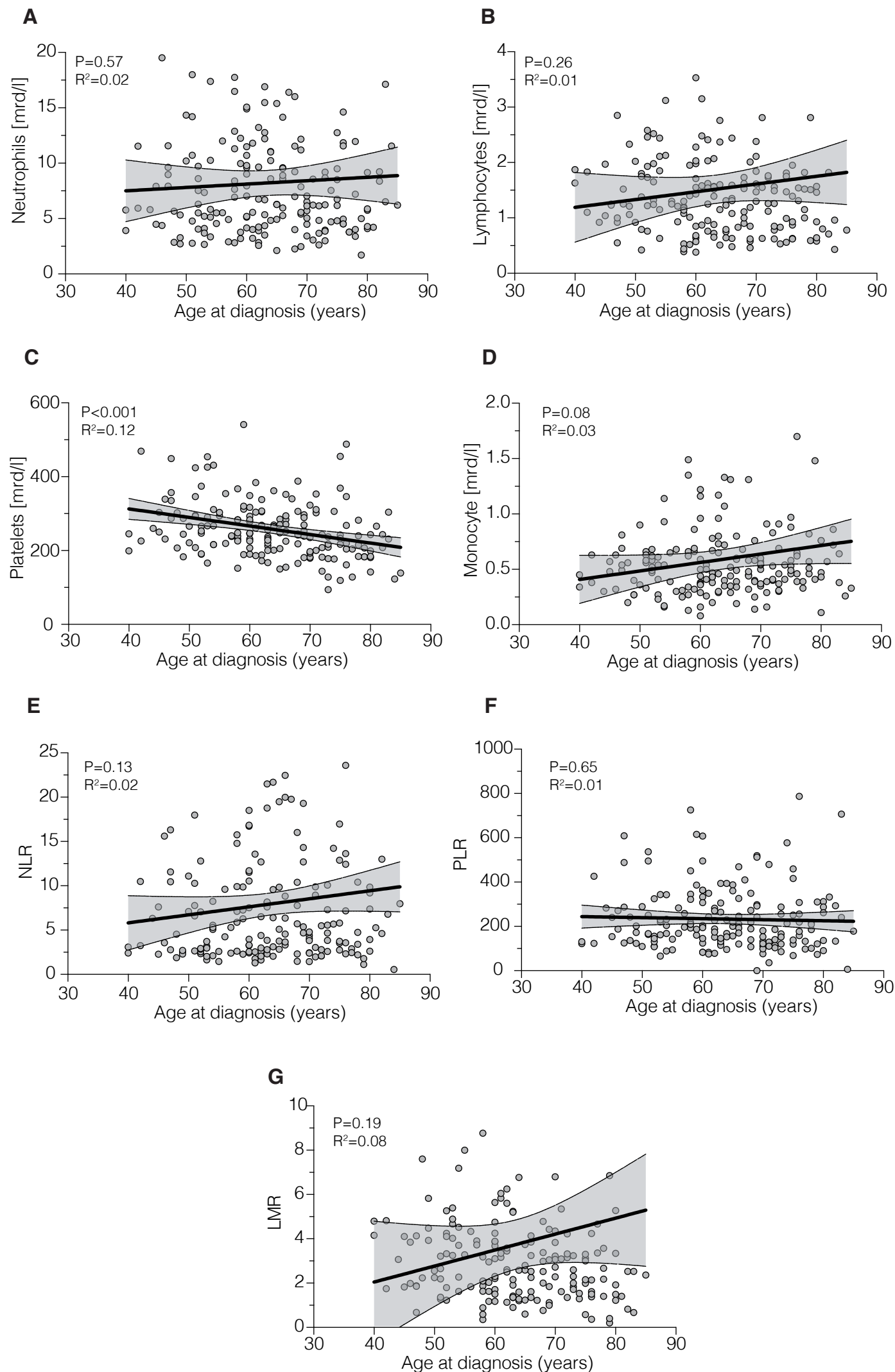

Supplement: Supplementary file 3 — Figure S3. Peripheral immune cell counts and composite scores correlated with the age of patients at the time of initial diagnosis. LMR, lymphocyte–monocyte ratio; NLR, neutrophil–lymphocyte ratio; PLR, platelet–lymphocyte ratio. [file BPA-35-e13334-s009.pdf]

**Supplementary Figure 4**

**A**

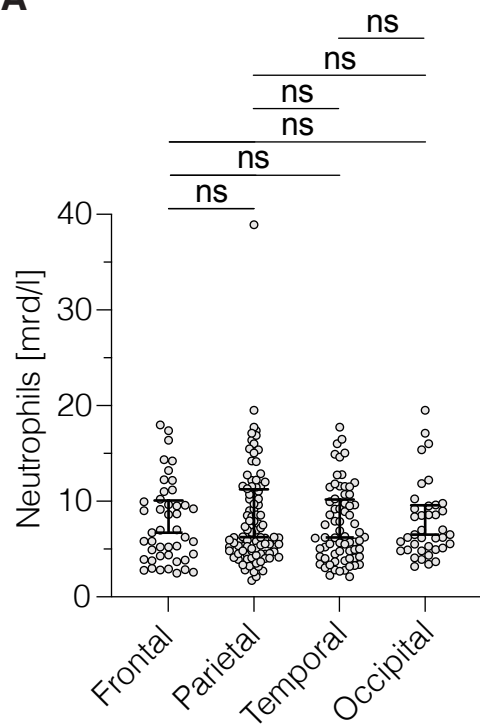

**B**

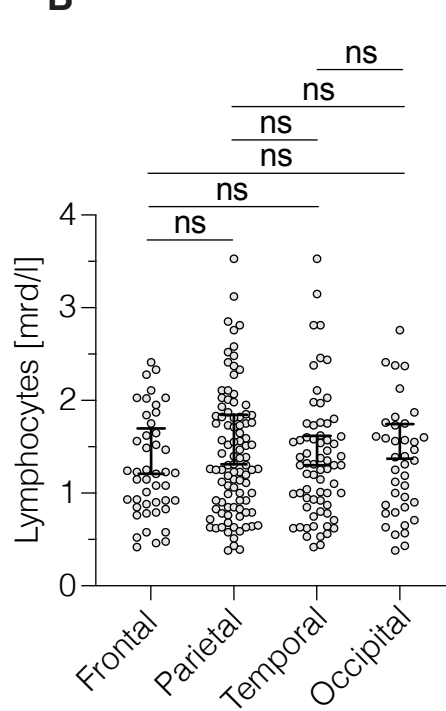

**C**

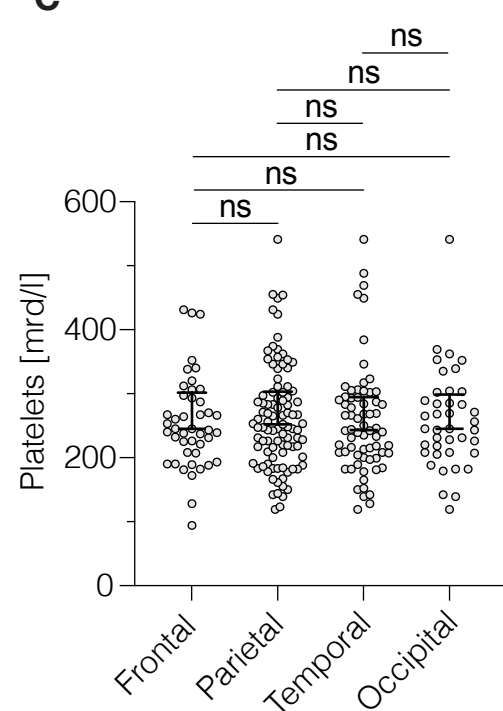

**C**

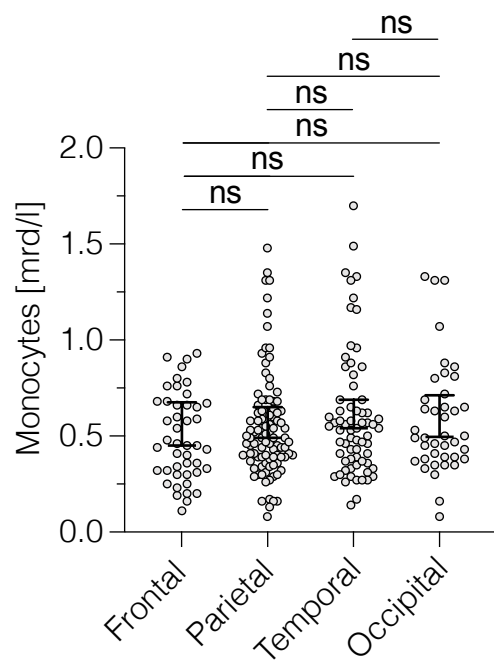

**D**

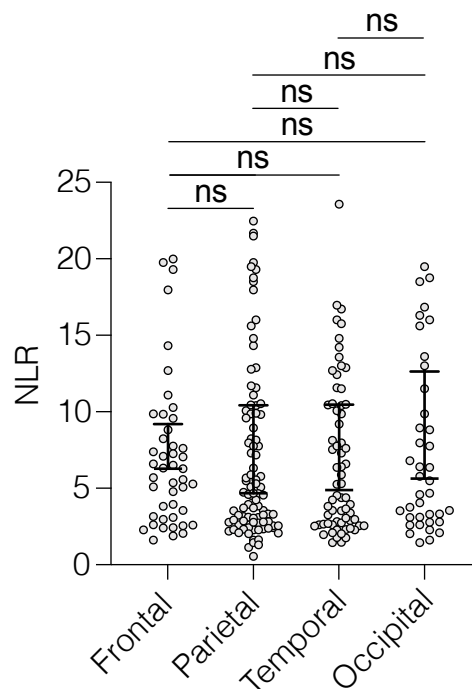

**E**

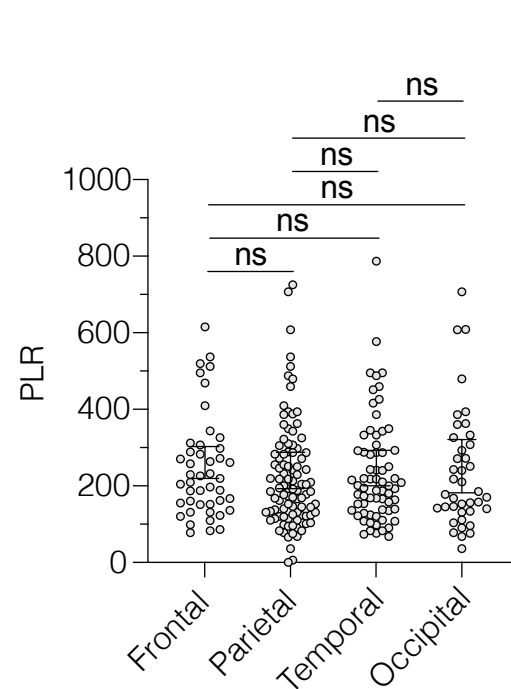

**F**

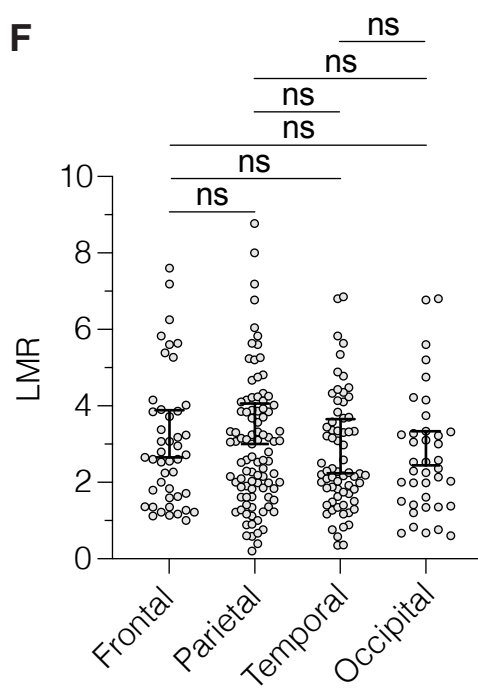

Supplement: Supplementary file 4 — Figure S4. Peripheral immune cell counts and composite scores correlated with the tumor location. LMR, lymphocyte–monocyte ratio; NLR, neutrophil–lymphocyte ratio; PLR, platelet–lymphocyte ratio. [file BPA-35-e13334-s005.pdf]

## Supplementary Figure 5

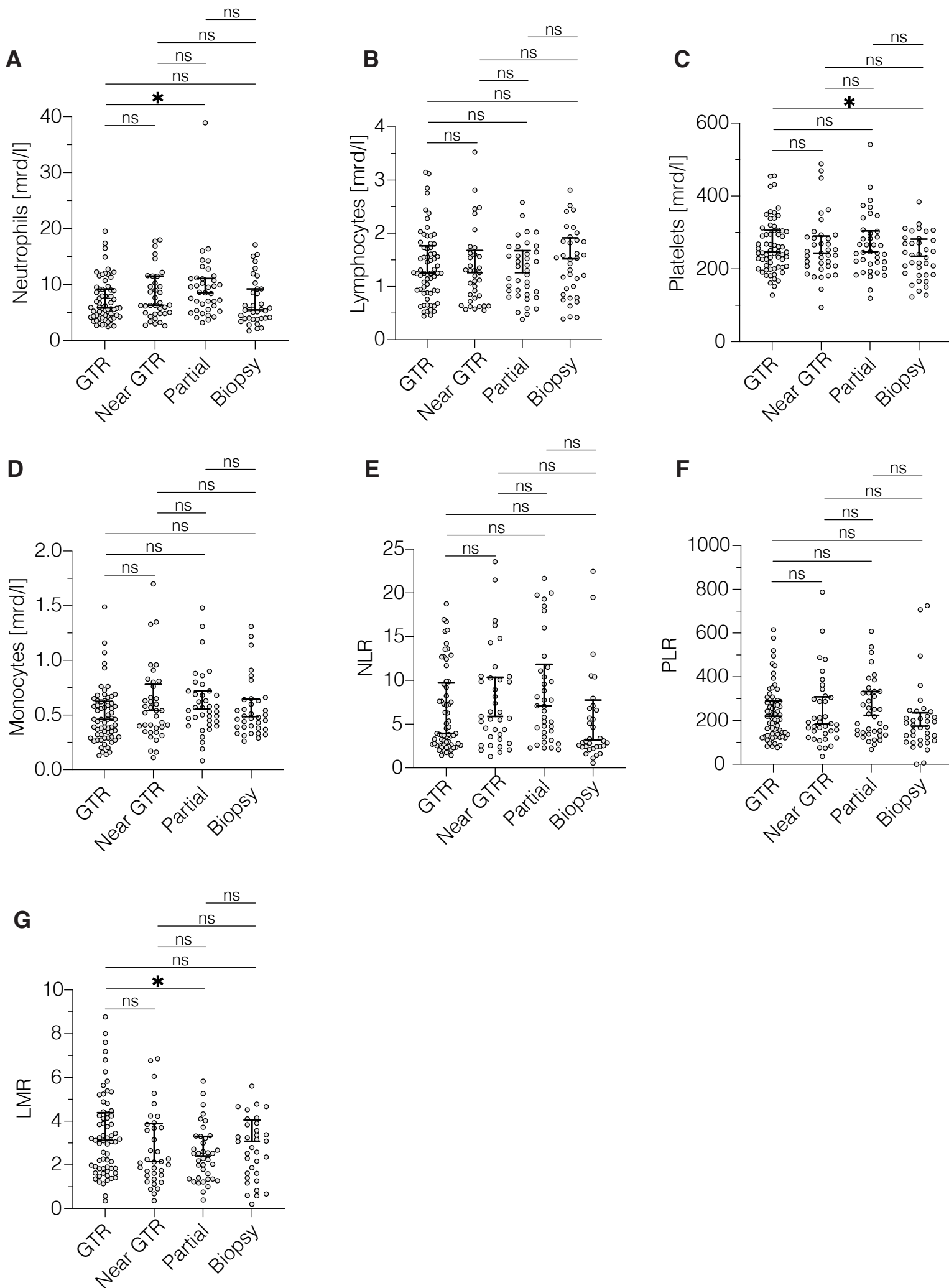

Supplement: Supplementary file 5 — Figure S5. Peripheral immune cell counts and composite scores correlated with the extent of surgery. LMR, lymphocyte–monocyte ratio; NLR, neutrophil–lymphocyte ratio; PLR, platelet–lymphocyte ratio. [file BPA-35-e13334-s007.pdf]

# Supplementary Figure 6

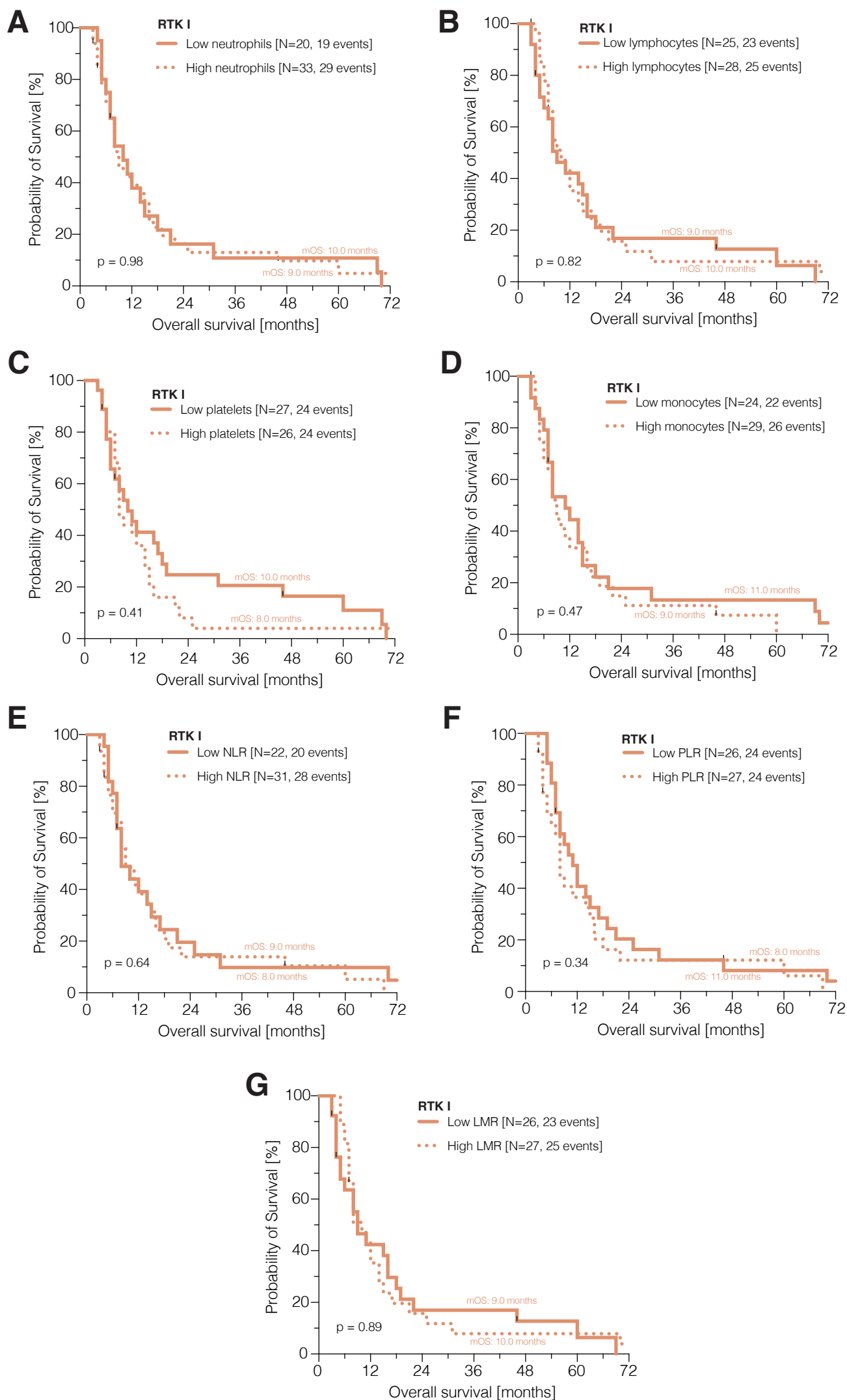

Supplement: Supplementary file 6 — Figure S6. Survival analysis on patients with newly diagnosed glioblastoma of the receptor tyrosine kinase I (RTK I) subgroup. [file BPA-35-e13334-s004.pdf]

# Supplementary Figure 7

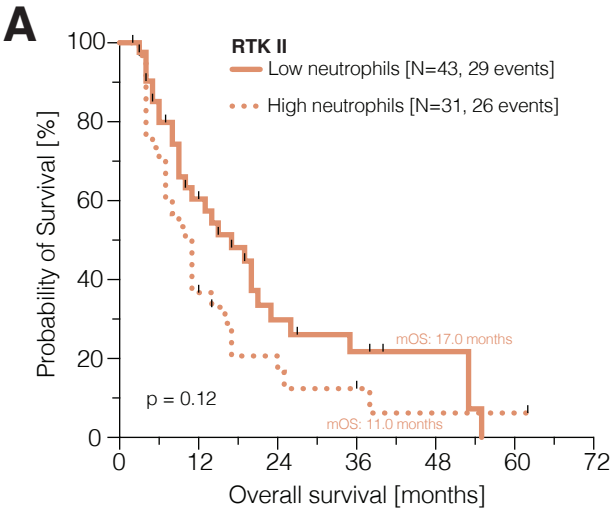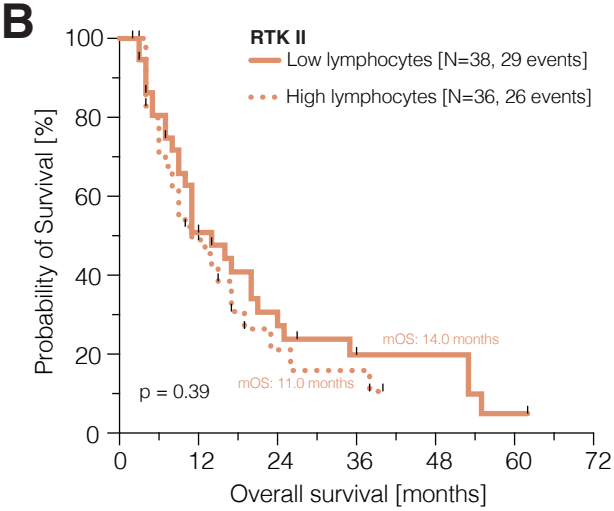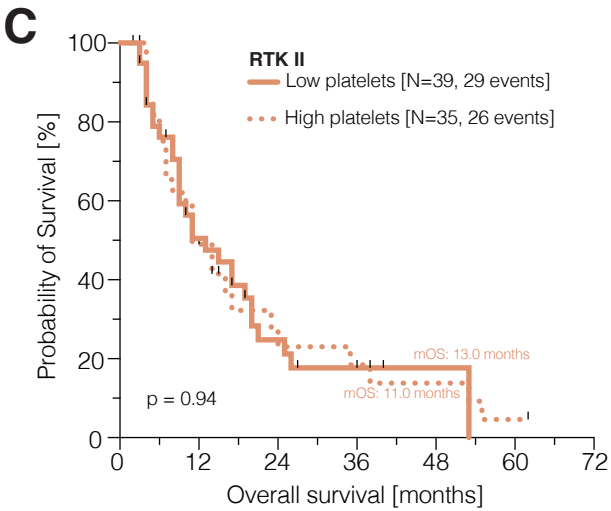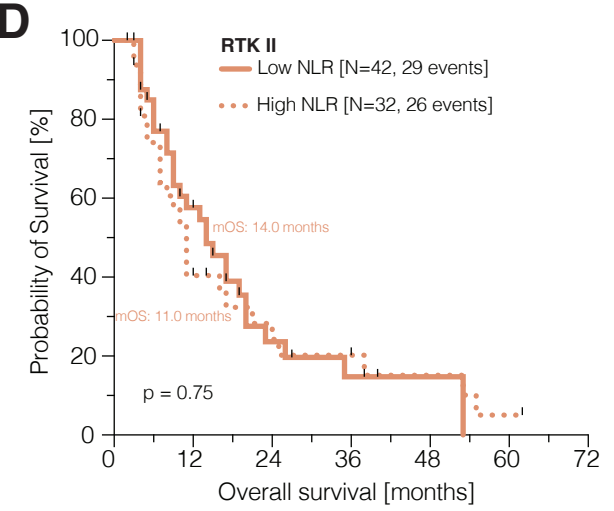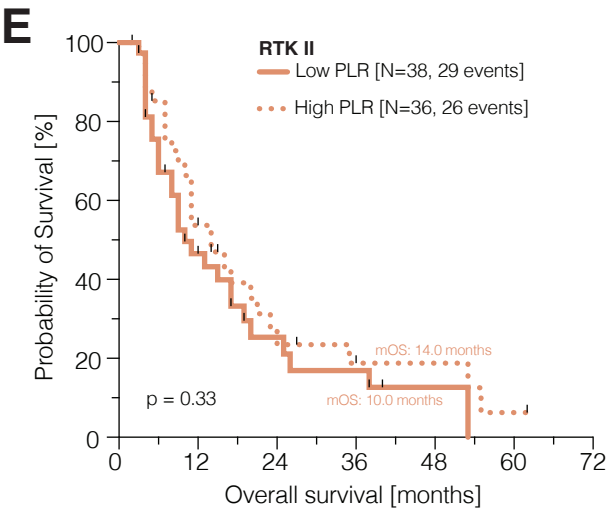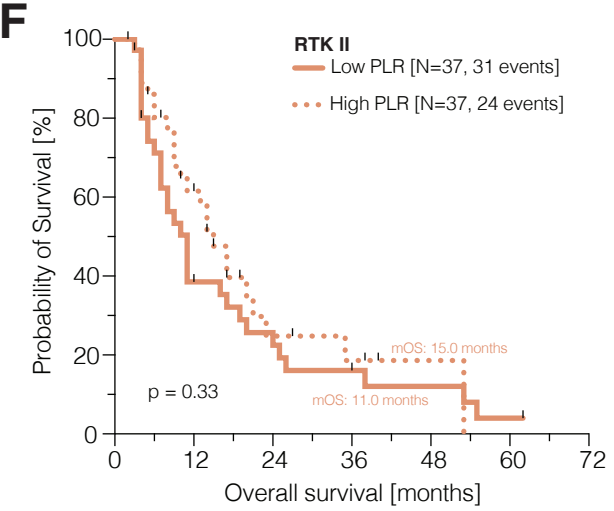

Supplement: Supplementary file 7 — Figure S7. Survival analysis on patients with newly diagnosed glioblastoma of the receptor tyrosine kinase II (RTK II) subgroup. [file BPA-35-e13334-s002.pdf]

# Supplementary Figure 8

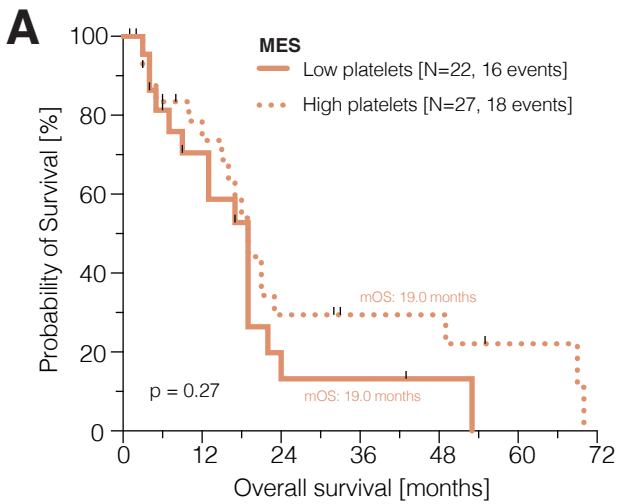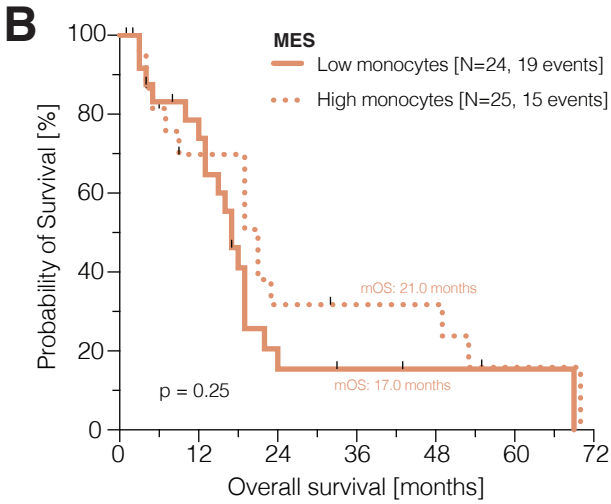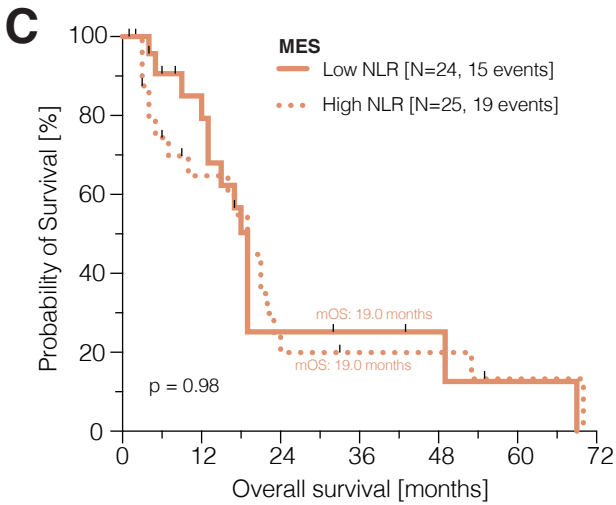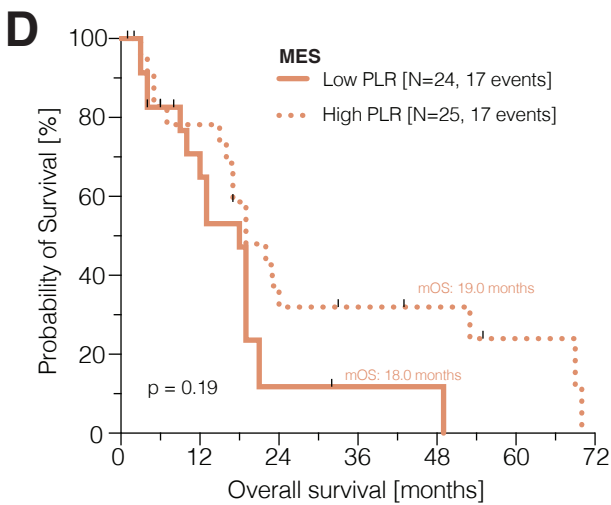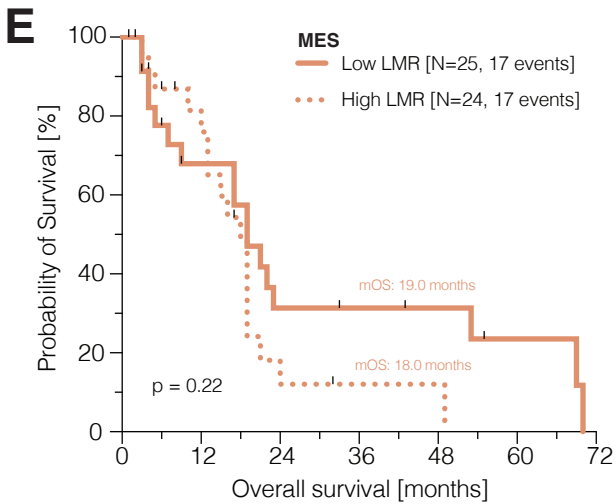

Supplement: Supplementary file 8 — Figure S8. Survival analysis on patients with newly diagnosed glioblastoma of the mesenchymal I (MES) subgroup. [file BPA-35-e13334-s010.pdf]

# Supplementary Figure 9

A

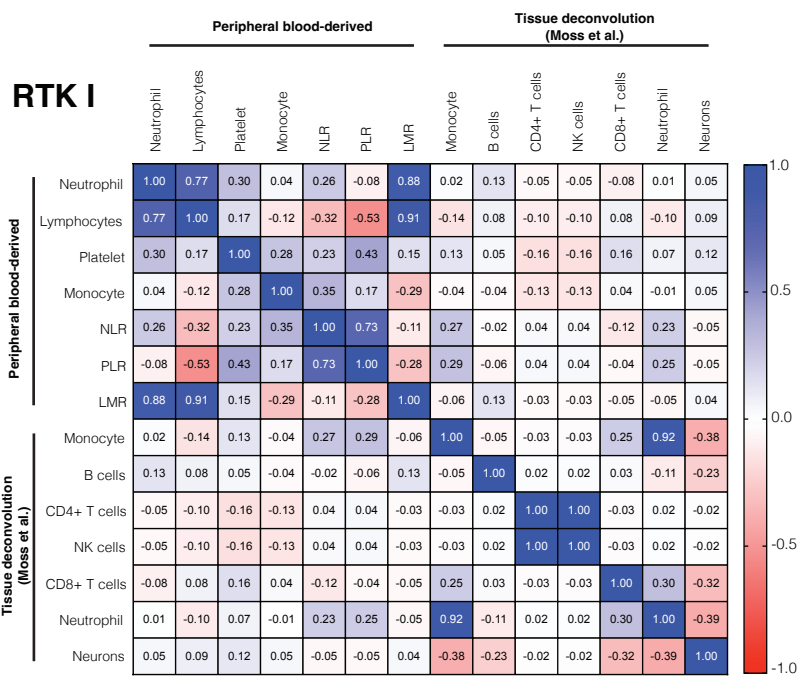

B

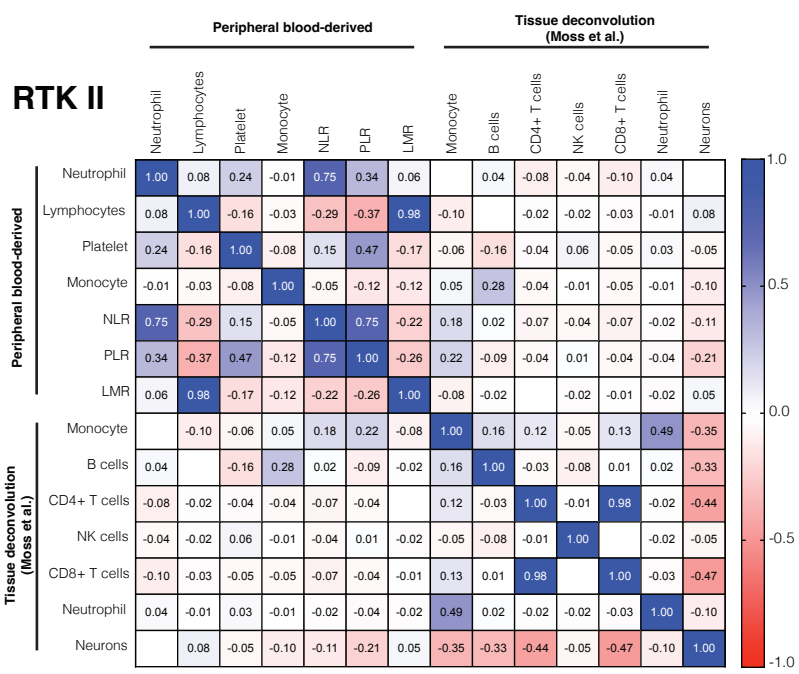

C

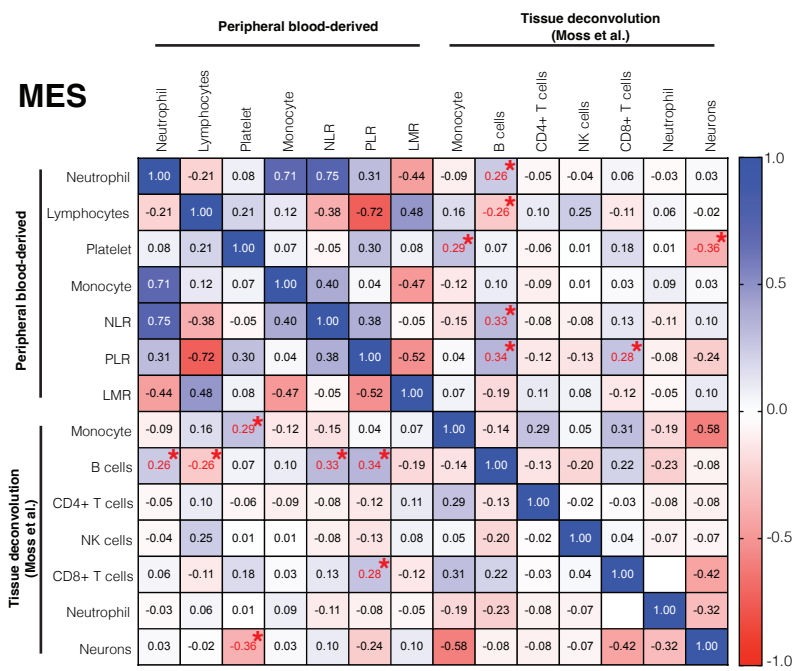

Supplement: Supplementary file 9 — Figure S9. Correlation matrix showing association between peripheral immune blood counts and composites scores with immune cells of matched tissue using deconvolution (Moss et al.) in the DNA methylation subgroup (A) RTK I, (B) RTK II, and (C) MES. *p < 0.05. [file BPA-35-e13334-s011.pdf]
