# Supplementary material for: Peripheral blood‐derived immune cell counts as prognostic indicators and their relationship with DNA methylation subclasses in glioblastoma patients
Source: Brain Pathol. 2025 Feb 3;35(4):e13334. doi: 10.1111/bpa.13334 (PMC12145900; doi:10.1111/bpa.13334)
Supplement: Supplementary file 10 — Table S1. Basic characteristics of the study population. LMR: lymphocyte–monocyte ratio; MGMT, O6‐methylguanine‐DNA‐methyltransferase; NLR, neutrophil–lymphocyte ratio; PLR, platelet–lymphocyte ratio; SD, standard deviation. [file BPA-35-e13334-s003.docx]

**Supplementary table 1:**

| **Characteristic** | **N=176** |
| --- | --- |
| Age, mean (SD) | 63.6 (10.5) |
| Sex, n (%) |  |
| Female | 71 (40.3) |
| Male | 105 (59.7) |
| Body mass index, mean (SD) | 26.4 (4.3) |
| Preoperative Karnofsky score, mean (SD) | 82.3 (12.8) |
| Preoperative NANO score, mean (SD) | 2.1 (2.2) |
| Location, n (%) |  |
| Frontal | 45 (25.6) |
| Parietal | 97 (55.1) |
| Temporal | 69 (39.2) |
| Occipital | 40 (22.7) |
| Eloquent | 120 (68.2) |
| Hemisphere, n (%) |  |
| Left | 89 (50.6) |
| Right | 73 (41.5) |
| Both | 14 (7.9) |
| Extent of resection, n (%) |  |
| Gross total | 65 (36.9) |
| Near gross total | 37 (21.0) |
| Partial | 38 (21.6) |
| Biopsy | 36 (20.5) |
| Preoperative neutrophil count, median (IQR) | 6.3 (4.5-10.2) |
| Preoperative lymphocyte count, median (IQR) | 1.3 (0.9-1.8) |
| Preoperative monocyte count, median (IQR) | 0.5 (0.4-0.7) |
| Preoperative platelet count, median (IQR) | 244.5 (205-296) |
| Preoperative NLR, median (IQR) | 5.3 (2.8-10.1) |
| Preoperative PLR, median (IQR) | 195.5 (132-292) |
| Preoperative LMR, median (IQR) | 2.6 (1.6-3.9) |
| *MGMT* promoter methylation status, n (%) |  |
| Non-methylated | 94 (53.4) |
| Methylated | 82 (46.6) |
| Ki67 index, mean (SD) | 26.4 (14.8) |
| Nuclear p53 accumulation, mean (SD) | 19.6 (22.5) |
| Karnofsky score prior adjuvant treatment, mean (SD) | 82.8 (15.4) |
| Days of blood draw before surgery, median (IQR) | 1 (0-3) |
| Daily dose of dexamethasone at time of surgery, [mg], median (IQR) | 6 (0-16) |
