# Supplementary material for: Peripheral blood‐derived immune cell counts as prognostic indicators and their relationship with DNA methylation subclasses in glioblastoma patients
Source: Brain Pathol. 2025 Feb 3;35(4):e13334. doi: 10.1111/bpa.13334 (PMC12145900; doi:10.1111/bpa.13334)
Supplement: Supplementary file 11 — Table S2. Multivariate analysis was conducted to evaluate the impact of clinical variables on patients' overall survival. [file BPA-35-e13334-s013.docx]

**Supplementary Table 2**

| **Feature** | **Reference** | **Hazard ratio** | **Lower 95% CI** | **Higher 95% CI** | ***P* value** |
| --- | --- | --- | --- | --- | --- |
| Age | - | 0.99 | 0.98 | 1.02 | 0.87 |
| Dexamethasone | No usage | 1.45 | 0.83 | 2.37 | 0.16 |
| Extent of resection | Gross total resection | 1.71 | 1.18 | 2.51 | **<0.01** |
| MGMT status | Non-methylated | 0.59 | 0.42 | 0.85 | **<0.01** |
| Karnofsky prior treatment | >70 | 1.61 | 1.09 | 2.33 | **0.01** |
