# Supplementary material for: Peripheral blood‐derived immune cell counts as prognostic indicators and their relationship with DNA methylation subclasses in glioblastoma patients
Source: Brain Pathol. 2025 Feb 3;35(4):e13334. doi: 10.1111/bpa.13334 (PMC12145900; doi:10.1111/bpa.13334)
Supplement: Supplementary file 12 — Table S3. Cox univariate model to assess the correlation between continuous immune cell counts and patients' overall survival. [file BPA-35-e13334-s001.docx]

**Supplementary Table 3**

| **Feature** | **Hazard ratio** | **Lower 95% CI** | **Higher 95% CI** | ***P* value** |
| --- | --- | --- | --- | --- |
| Neutrophils | 0.98 | 0.91 | 1.02 | 0.56 |
| Lymphocytes | 1.06 | 0.81 | 1.20 | 0.53 |
| Platelets | 0.99 | 0.98 | 1.00 | 0.97 |
| Monocytes | 1.06 | 0.60 | 1.53 | 0.80 |
| NLR | 0.98 | 0.93 | 1.03 | 0.61 |
| PLR | 0.99 | 0.98 | 1.00 | 0.29 |
| LMR | 0.98 | 0.87 | 1.04 | 0.87 |
