# Supplementary material for: Peripheral blood‐derived immune cell counts as prognostic indicators and their relationship with DNA methylation subclasses in glioblastoma patients
Source: Brain Pathol. 2025 Feb 3;35(4):e13334. doi: 10.1111/bpa.13334 (PMC12145900; doi:10.1111/bpa.13334)
Supplement: Supplementary file 13 — Table S4. Cutoff values for each peripheral immune cell or composite score to stratify the study population into low or high counts. [file BPA-35-e13334-s006.docx]

**Supplementary Table 3**

| **Peripheral immune cell or composite score** | **Count** |
| --- | --- |
| **Neutrophils** |  |
| Low | < 6.3 |
| High | > 6.3 |
| **Lymphocytes** |  |
| Low | < 1.3 |
| High | > 1.3 |
| **Platelets** |  |
| Low | < 245 |
| High | > 245 |
| **Monocytes** |  |
| Low | < 0.5 |
| High | > 0.5 |
| **Neutrophil-lymphocyte ratio** |  |
| Low | < 5.29 |
| High | > 5.29 |
| **Platelet-lymphocyte ratio** |  |
| Low | < 196 |
| High | > 196 |
| **Lymphocyte-monocyte ratio** |  |
| Low | < 2.6 |
| High | > 2.6 |
